# Knowledge of and attitudes toward heel prick screening for sickle cell disease in Saint Lucia

**DOI:** 10.26633/RPSP.2017.70

**Published:** 2017-04-28

**Authors:** Sonia Alexander, Sharon Belmar-George, Alisha Eugene, Vanessa Elias

**Affiliations:** 1 Monroe College, Saint Lucia Campus Monroe College, Saint Lucia Campus Castries Saint Lucia Monroe College, Saint Lucia Campus, Castries, Saint Lucia.; 2 Ministry of Health and Wellness Ministry of Health and Wellness Castries Saint Lucia Ministry of Health and Wellness, Castries, Saint Lucia.; 3 Pan American Health Organization Pan American Health Organization Washington, D.C. United States of America Pan American Health Organization, Washington, D.C., United States of America.

**Keywords:** Anemia, sickle cell, health personnel, health communication, Saint Lucia, West Indies, Anemia de células falciformes, personal de salud, comunicación en salud, Santa Lucía, Indias Occidentales, Anemia falciforme, pessoal de saúde, comunicação em saúde, Santa Lúcia, Índias Ocidentais

## Abstract

**Objectives.:**

In the Caribbean country of Saint Lucia, umbilical-cord-blood screening for sickle cell disease (SCD) was the testing method that health care workers (HCWs) on the maternity wards of the hospitals preferred until the new heel prick (HP) testing method was introduced in the country in 2014. This SCD study sought to assess HCWs’ knowledge of and attitude toward HP screening and also determine new mothers’ favorability toward HP screening.

**Methods.:**

A total of 70 HCWs and 132 new mothers answered survey questionnaires in three hospitals. In addition, four focus group discussions were held, two with HCWs and two with the mothers.

**Results.:**

Among the HCWs interviewed, 85.7% of them had knowledge of the HP test. However, only 25.7% had attended training sessions on the procedure. Among the HCWs, 64.3% of them felt the HP test should be mandatory, 27.1% said it should not be mandatory, and 8.6% did not know if it should be mandatory. In their focus groups, the HCWs said they believed the mothers would accept the HP method. For their part, 22.0% of the mothers said they had heard about the HP test, and 63.6% reported knowing the reason why the baby would be tested. Further, 83.3% indicated that the test would be beneficial for the baby. In addition, 88.6% of the mothers said that more information on the HP test was needed. In their focus group discussions, the mothers said they were generally not concerned about the pain the heel prick method might cause the baby.

**Conclusions.:**

The HCWs’ knowledge of the HP screening method was high. The mothers trust HCWs, and the mothers would accept the HP procedure irrespective of their knowledge of the test and any discomfort associated with this screening method.

## RESUMEN

**Objetivo.:**

En el país caribeño de Santa Lucía, el hemocribado del cordón umbilical para la detección de la anemia de células falciformes o drepanocitosis era el método de tamizaje preferido por el personal de salud en las salas de maternidad de los hospitales hasta que se introdujo la nueva prueba del talón en el 2014. Este estudio sobre la anemia de células falciformes tuvo por objetivo evaluar el conocimiento y las actitudes del personal de salud hacia la prueba del talón, así como determinar si las madres tenían una actitud favorable hacia este método de tamizaje.

**Método.:**

Un total de 70 trabajadores de salud y 132 madres participaron en los cuestionarios realizados en tres hospitales. Además, se celebraron cuatro grupos de debate: dos con el personal de salud y dos con las madres.

**Resultados.:**

Del personal de salud entrevistado, 85,7% tenía conocimientos acerca de la prueba del talón. Sin embargo, únicamente 25,7% había asistido a sesiones de capacitación sobre el procedimiento. Además, 64,3% del personal de salud opinaba que la prueba debía ser obligatoria, 27,1% afirmaba que no debía ser obligatoria y 8,6% manifestó que no lo sabía. En los grupos de debate, el personal de salud manifestó su creencia de que las madres aceptarían este método de tamizaje. Por su parte, 22,0% de las madres afirmaron conocer la prueba del talón y 63,6% manifestó conocer los motivos por los que se sometería al bebé a dicha prueba. Asimismo, 83,3% de las madres señaló que la prueba sería beneficiosa para el bebé. Además, 88,6% de las madres indicó que era necesaria más información sobre la prueba del talón. En sus grupos de debate, las madres expresaron que, por lo general, no les preocupaba el dolor que pudiese suponer para el bebé la prueba del pinchazo en el talón.

**Conclusiones.:**

El personal de salud tiene un nivel alto de conocimiento acerca de la prueba del talón. Las madres confían en el personal de salud, y aceptarían este procedimiento independientemente de sus conocimientos sobre la prueba y cualquier malestar asociado a este método de tamizaje.

## RESUMO

**Objetivos.:**

No país caribenho de Santa Lúcia, o exame de sangue de cordão umbilical para detecção da doença falciforme (DF) era o método de triagem preferido pelos profissionais da saúde atuantes em maternidades, até a introdução do “teste do pezinho” (TP) no país em 2014. Este estudo sobre DF buscou avaliar o conhecimento e atitudes de profissionais da saúde em relação à triagem pelo TP e também determinar a favorabilidade das novas mães em direção à triagem de TP.

**Métodos.:**

Setenta profissionais da saúde e 132 novas mães responderam a questionários de pesquisa em três hospitais. Além disso, foram realizadas quatro discussões com grupos focais: dois de profissionais da saúde e dois de mães.

**Resultados.:**

Entre os profissionais da saúde entrevistados, 85,7% tinham conhecimento acerca do TP. Porém, somente 25,7% haviam comparecido às sessões de capacitação sobre o procedimento. Entre os profissionais da saúde, 64,3% opinaram que o TP deveria ser obrigatório, 27,1% disseram que não deveria ser obrigatório e 8,6% não sabiam se deveria ser obrigatório. Em seus grupos focais, os profissionais da saúde disseram acreditar que as mães aceitariam o método do TP. Por sua vez, 22,0% das mães disseram ter ouvido falar sobre o TP, e 63,6% relataram saber o motivo pelo qual o exame seria realizado; ademais, 83,3% indicaram que o exame seria benéfico para o bebê. Além disso, 88,6% das mães disseram que precisavam de maiores informações sobre o TP. Em seus grupos focais, as mães disseram que, em geral, não estavam preocupadas com dor que o teste do pezinho poderia causar ao bebê.

**Conclusões.:**

Os profissionais da saúde apresentaram alto nível de conhecimento acerca do método de triagem pelo TP. As mães confiam nos profissionais da saúde, e aceitariam o procedimento de TP independentemente de seu conhecimento acerca do teste e de qualquer desconforto associado a este método de triagem.

Sickle cell disease (SCD) describes a group of inherited red blood cell disorders in which abnormal hemoglobin, called hemoglobin S or sickle hemoglobin, forms in red blood cells (1). Infants born with SCD are at a very high risk of morbidity and mortality, and early detection allows for prompt intervention (1).

An estimated 300 000 infants each year are born worldwide with SCD, with the prevalence being highest in sub-Saharan Africa and in the Caribbean (2). The World Health Organization estimates that 70% of SCD deaths in Africa are preventable with simple, cost-effective interventions such as early identification of SCD patients by newborn screening and the provision of comprehensive care (3).

The country of Saint Lucia is located in the eastern Caribbean and is a member of the Organization of Eastern Caribbean States (OECS). Its two closest neighbors are Martinique and Saint Vincent and the Grenadines. The population of Saint Lucia as of mid-year 2012 was 169 115. The country’s population is predominantly of African descent. The total fertility rate per woman was 1.5 in 2011 and 2012 (4).

According to a report on the Saint Lucia SCD program prepared by the country’s Ministry of Health and Wellness (MOHW) in 2014, SCD occurs, on average, in every 1 of 150 infant births (5). In 1992, Saint Lucia had introduced an umbilical-cord-blood screening initiative for SCD, which it integrated into the Community Child Health Service of the MOHW, with the goal of working toward universal screening. This initiative was supported by the St. Lucia Sickle Cell Association, a local nongovernmental organization.

Without screening, infants with SCD are at risk of complications. The two most common are vaso-occlusive pain crisis and acute chest syndrome, which is a lung injury syndrome (6). In order to strengthen the Saint Lucia SCD program, the MOHW began procuring the pneumococcal vaccines used in the treatment of SCD. These vaccines are distributed to pediatricians in the public and private sectors in the country for the treatment of children free of cost. Despite SCD being the most prevalent genetic disorder in Saint Lucia as of 2010, screening was only being done with approximately 80% of babies (5).

The 2014 MOHW audit reported that of 36 253 babies checked in Saint Lucia, 3 146 of them (9.0%) had sickle cell trait, where babies inherit one sickle cell gene from one parent and one normal gene from the other parent. A total of 180 babies (0.5%) had homozygous SCD, indicating that the infants had inherited two sickle cell genes, one from each parent. Hemoglobin sickle cell disease was present in 59 of the babies (0.1%) (5).

One alternative to SCD cord blood screening is the heel prick (HP) filter paper method. In comparison to the HP method, cord blood screening has unacceptably high false-positive and false-negative rates due to contamination with maternal blood, as well as method-specific errors. The procedures for HP blood sample collection on filter paper, processing, and storage have been fairly standardized and are easily adaptable.

In 2010, a pilot project involving Saint Lucia and Jamaica focused on improving newborn screening for SCD in Saint Lucia. The project aimed to centralize the SCD newborn screening tests, through the Tropical Medicine Research Unit in Kingston, Jamaica. The pilot used a dry filter paper blood spot method, with samples obtained directly from the newborn by HP and then sent to Jamaica for analysis.

When considering a new process like this for SCD screening, perceptions of the new procedure can be pivotal in determining the strategy for seeking authorization from MOHW officials and buy-in from health care workers (HCWs) and mothers and other caretakers. This is due to fact that the process involves a heel prick to the newborn, which is considered to be more invasive than the traditional cord blood process. In a study conducted in the United Kingdom, mothers’ attitudes toward newborn blood spot screening depended on their level of trust of midwives (7).

The objectives of this study were to assess the knowledge of and attitude toward heel prick screening for SCD on the part of HCWs in Saint Lucia, to determine the level of mothers’ acceptability of the heel stick screening, and to utilize the data generated to guide policy and program development.

## MATERIALS AND METHODS

This work was developed as part of an initiative called Improving Program Implementation through Embedded Knowledge Translation and Research (iPIER). The initiative was developed by the Alliance for Health Policy and Systems Research in collaboration with the Pan American Health Organization (PAHO). The iPIER model (http://www.who.int/alliance-hpsr/projects/ir_projects_ipier/en/) places program implementers in the center of an investigation in order to learn about failures of the health system that create barriers to implementation and also to identify solutions to those barriers.

**FIGURE 1.**
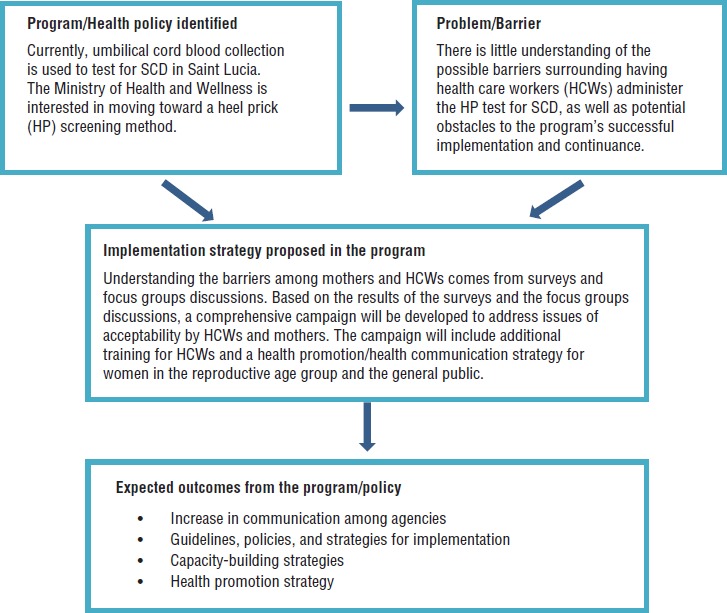
Flowchart of the sickle cell disease (SCD) screening-method project protocol used in Saint Lucia, 2015–2016

Figure 1 is a flowchart showing the protocol used for this study.

### Setting

The Government of Saint Lucia provides primary and secondary health care services in nine health regions. For the provision of primary health care, there are 33 public sector wellness centers, 1 polyclinic, and 2 small district hospitals. Secondary health services are also provided through three hospitals. Victoria Hospital, in the city of Castries, is the main hospital, and St. Jude hospital is located in the south of the country. Tapion Hospital is a small private hospital in the city of Castries. Of the births in Saint Lucia, 98% of them occur in these three hospitals. For this study, mothers and HCWs were surveyed at the three hospitals.

### Study design

This cross-sectional descriptive study was conducted from December 2015 to February 2016. In this multisite national study, a convenience sampling method was utilized at the three hospitals, among women who gave birth as well as among HCWs (doctors and nurses) who were present on the maternity wards and consented to participate.

### Sample

The mother-participants were selected from eligible women on the maternity wards. From a selected start day in December 2015, all eligible new mothers who were present on the wards were recruited on alternate days, until the sample size of 132 women was achieved in February 2016.

The desired sample size was calculated by first taking the mean number of live births in Saint Lucia over the preceding five years, which was 2 100 per year. We assumed a level of the mothers’ acceptance of the new-born HP screening of 90%, based on a literature review. Using a 95% confidence interval and a 5% attrition rate, we calculated the sample size to be 140; however, the final number of women enrolled in the survey was 132.

Due to the limited number of HCWs on the maternity wards, we recruited all the doctors and nurses on the maternity wards at the three hospitals who were present on the alternate survey days during the study period.

### Exclusion criteria

Excluded from the study were HCWs who did not provide care to women on the maternity wards and women who had not delivered at the time of data collection. Also excluded from the study were women whose babies were ill or stillborn, as well as girls 16 years of age or younger.

### Measures

To collect information, study interviewers used two standard questionnaires, one for the HCWs and one for the mothers. The questionnaires were divided into four sections: a) identification information; b) demographic information; c) knowledge of the HP method; and d) attitude towards the HP screening method.

Four focus group discussions (FGDs) were also conducted to collect information, two with HCWs and two with the new mothers. A consultant hired by the MOHW developed two open-ended interview guides for those focus group discussions.

### Procedures

The work team consisted of the consultant hired by the MOHW, three data collectors, and two data entry clerks. The consultant trained the data collectors and data entry clerks on the proper techniques needed for the study.

A steering committee was appointed by the MOHW to oversee the project. The steering committee was led by the Medical Officer of Health in the MOHW, who is responsible for the supervision and operation of primary care and public health services within the MOHW.

#### Focus group discussions.

For the FGDs, the consultant coordinated the sessions, with assistance from two of the data collectors. A total of 18 HCWs and 17 mothers participated in the FGDs. Those sessions lasted from 45 to 60 minutes and were recorded electronically, and with handwritten notes taken down by a data collector. The day after each FGD, the consultant and the data collector used the notes and the recording to complete the narrative of the session in a report.

#### Surveys.

All HCWs present on the maternity wards on the designated survey days were invited to participate in the study, and the same was done with all the women who had delivered a healthy baby. The mothers were recruited on the same day they delivered their babies or on the following day. The HCWs were recruited during work time or when the shift ended.

### Analysis

The quantitative and qualitative analysis was completed by the consultant. The quantitative analysis was done using SPSS software and presented as descriptive data. Relevant statistical tests of significance were done for each variable observed. Double entry was done using Epi Info software to check for completeness and errors in data entry.

For the qualitative analysis of the FGD data, content analysis was done, using the FGD reports. The consultant grouped the data according to the key themes that had emerged in the sessions. The consultant further segmented the findings into knowledge and attitudes categories and assessed the information for meaningfulness and for similarities between the mothers and the HCWs.

The PAHO Ethical Review Board and the Saint Lucia Medical and Dental Council granted ethical approval for the study. Written informed consent was obtained from the study participants (HCWs and mothers) after explanation of the study protocol, including potential risks and benefits. The participants were informed that there were no risks associated with the study and that their decision to participate was voluntary. In addition, the HCWs were informed that participating would not affect their jobs in any way. The consultant also informed the HCWs and the new mothers that all the information they provided would be anonymized, that they would not be able to be identified, and that the information would be kept in the strictest confidence. The mothers were further informed that if they refused to participate, it would not affect their care at the hospital or other health facilities in any way.

## RESULTS

### Health care workers

#### Survey.

There were a total of 80 HCWs employed on the maternity wards at the three hospitals, of which 70 of them were interviewed. Of those 70, 34 of them (48.6%) were employed with Victoria hospital, 19 (27.1%) with St. Jude, and 17 (24.3%) with Tapion.

Of the 70 HCWs, 35 of them (50.0%) were 35 years old or over; 19 (27.1%) were between 30 and 34; 12 (17.1%) were between 25 and 29; and 4 (5.9%) were between 20 and 24. Occupational information was obtained for 64 HCWs: doctors accounted for 10 of the 64 (15.6%) of the respondents and nurses/nurse midwives for 54 (84.4%).

Overall, out of the cohort of 70 HCWs who were interviewed, 60 of them (85.7%) had knowledge of the HP test. However, only 18 of the 70 (25.7%) had attended training sessions on the procedure. Nevertheless, 45 of the 70 HCWs (64.3%) felt the HP test should be mandatory, 19 (27.1%) felt it should not be mandatory, and 6 (8.6%) did not know if it should be mandatory. Of the 70, 63 of them (90.0%) would encourage the mothers to allow the test to be done for their babies. In terms of the difficulty of collecting the HP sample versus the cord-blood samples, 25 of the 70 (35.7%) felt that collecting the HP sample was more tedious. In addition, 41 of the 70 (58.6%) reported it was faster to obtain the test results with the cord-blood samples than with the HP samples.

#### Focus groups.

Two focus group discussions were held with HCWs on the maternity wards, with a total of 18 participants. In terms of knowledge and training, all the HCWs reported having knowledge of what SCD is. In addition, all of the 18 also said they had had a previous introduction to the HP method, but only 4 of the 18 had been trained by medical personnel on using this method. Rather than by participating in a formal training course, one HCW reported being trained by “reading the chart,” which was displayed on the wall of the maternity ward and outlined the process for obtaining a sample using the HP method.

With respect to the issue of acceptance, the HCWs said they believed that “the mothers will accept the HP test without question.” To encourage the dissemination of SCD information to mothers in Saint Lucia, HCWs felt that personnel of the MOHW should consider “more public awareness, teaching, and informing mothers about SCD.” The HCWs also suggested that the discussion with pregnant women and other caretakers about SCD and the HP method should begin in the antenatal clinics.

Concerning needed supplies, the HCWs expressed concerns about the inconsistent availability of the “right tools and materials,” such as lancets and the filter paper for blood spot collection. One HCW emphasized, **“**We do not have an issue with the HP, our issue is the availability of the materials, especially the lancets.”

With regard to their attitude towards SCD screening, all the HCW focus group participants felt that SCD testing is essential for early diagnosis and treatment. One HCW offered this summary: “The burden on the heath system would be a lot less. You have more persons able to manage their condition if it is diagnosed earlier.”

On the subject of cord blood versus HP screening, the HCWs viewed the cord blood screening method more favorably and indicated that it is likely that more babies would be screened through that approach. As one person said, “It takes less time, and you are likely not to miss a client.”

### Mothers

#### Survey.

A total of 132 new mothers were interviewed. Of those women, 105 of them (79.5%) delivered at Victoria Hospital, 26 (19.7%) at St. Jude Hospital, and 1 (0.8%) at Tapion Hospital. In terms of age, 21 of the women (15.9%) were between the age of 17 and 19; 28 (21.2%) were between 20 and 24; 29 (22.0%) were between 25 and 29; 30 (22.7%) were between 30 and 34; and 24 (18.2%) were 35 or older.

With respect to employment status, 45 (34.1%) were employed full time; 1 woman (0.8%) was employed part time; 14 (10.6%) were self-employed; and 72 (54.5%) were unemployed. A total of 14 (10.6%) reported attending primary school as their highest level of education, and 118 (89.4%) had attended secondary school.

All 132 of the mothers answered all the questions on the survey. With respect to the HP test, 29 of the mothers (22.0%) said they had heard about the test, and 84 (63.6%) reported knowing the reason why the babies would be tested. Of the mothers, 99 (75.0%) reported that the test cannot prevent diseases, and 78 (59.1%) responded positively when asked if they knew any of the diseases that the test was used to screen for. Further, 83.3% said the test would be beneficial for the baby, and 89.4% stated early diagnosis can help if a genetic disease is diagnosed. Among the new mothers, there was broad support for using the HP test for SCD screening; 130 women (98.5%) endorsed its use for screening all babies. Among the cohort of mothers, 79 (59.8%) felt that the HP test would not be painful for the babies.

**FIGURE 2.**
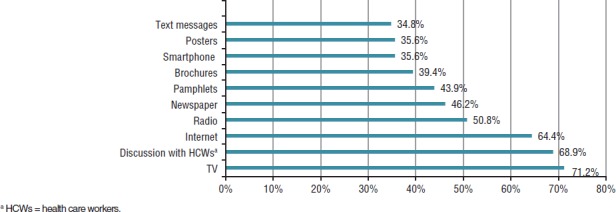
New mothers’ preferred channels for receiving additional information on the heel prick screening test for sickle cell disease, Saint Lucia, 2015–2016

^a^ HCWs = health care workers.

In their survey responses, the mothers overwhelmingly said that additional information on the HP test was needed, with 117 of them (88.6%) expressing that view. The women suggested that the best channels for them to receive additional information on the test would be television, discussions with health care workers, the Internet, and radio (Figure 2).

#### Focus groups.

In the two FGDs for the mothers, 17 women participated. One of the key findings from these sessions was that the mothers were generally not concerned about the pain the HP method might cause the baby. One mother said, “Whether it is painful or not … it is better, it is good for the child’s health.” They reported that they would “just have to comfort the babies” during and after the testing. Overall, a high level of acceptance for the HP test was expressed in the FGDs. One woman remarked, “They don’t have to ask to do it if it is to protect the child and it is important.”

Currently, information on SCD is shared with mothers in the antenatal clinics in the first trimester and on the maternity wards immediately after delivery. Midwives provide the information at the antenatal clinics, and doctors and midwives do that on the maternity wards.

All the mothers in the FGDs agreed that HCWs need to provide them with more information so they can make an informed decision about testing for SCD. The mothers wanted to be informed on the procedure and the policy on the HP test for SCD. One said that “they should inform you before you give birth what will happen to your baby. I don’t want to know after, I want to know before.”

## DISCUSSION

This study was conducted among two well-defined groups: HCWs and women who delivered their babies on the maternity wards of the three main hospitals in Saint Lucia. Enrollment was based on a convenience sample, and informed consent was obtained. The main objective of this study was to explicate the knowledge and attitudes of both the HCWs and the mothers regarding the HP screening method for SCD.

This is the first study in Saint Lucia to document the knowledge and attitudes of HCWs and women of reproductive age on SCD. Up to this point, little information has been available in the country to inform decisions and policies on SCD screening tests.

These findings must be assessed in the context of the study’s limitations, including the environment in which the data were collected. The women were in the hospital, and they had delivered either on the same day or the day before the interview was conducted. While most of the mothers were very cooperative, many were visibly in pain or were attending to their babies. For their part, the HCWs were extremely busy, given the human resource constraints that are pervasive in the country’s health care system.

This study indicates that the HCWs’ knowledge on the SCD HP test was high: 85.7% of them knew about the test. As could be expected, HCWs undoubtedly understood the implications of not having the test done. From the results of the FGDs, it’s clear that the HCWs’ knowledge of the diseases that the HP test can be used to detect was understandably superior to that of the mothers. Although only 22.0% of the mothers reported that they had heard about the HP test, 63.6% of them reported knowing the reason why the baby would be tested. This finding may be partly attributed to the fact that the women receive some information on SCD and the screening methods in the antenatal clinics and on the maternity wards.

The results indicated that both HCWs and mothers supported population-based testing and screening at birth for SCD. This is a strong indication of the acceptance of the HP test, but there is still a need to consider the issue of consent or of having a distinct choice. While the mothers know little about the HP test, they are in favor of mandatory testing. Similarly, the HCWs felt the test should be given even without consent. The perceived painfulness of the test was not a major contention nor was it an important deterrent to the mothers, given that 59.8% of the mothers believed that the test would not be painful. They maintained that they could comfort the baby during the test or after, and that that would make the test acceptable to them. This indicated that the mothers have a high level of trust in the HCWs, who are influential in the decisions the mothers make. For the mothers to make an informed choice, HCWs have to ensure that the information they give is correct, is understood, and is provided through the channels that are easily accessible for the mothers.

Both the HCWs and the mothers felt that information was needed to better inform the mothers on making a choice on whether to accept the test or not. This study is consistent with other research findings that suggest that having information is crucial to the decision-making process. In one study conducted in the United Kingdom (7), researchers found evidence that the mothers wished to have midwives inform them about the HP screening.

Training HCWs to not only conduct the test but to also provide information to the mothers is necessary. In a study on SCD and neonatal screening conducted in the Netherlands, the researchers concluded, “Among primary care providers, there is a lack of knowledge about both SCD itself as well as about their role in the process” (8). In the Saint Lucian context, 88.6% of the mothers said they needed additional information. The three information channels they most preferred were television, discussions with HCWs, and the Internet. While those first two channels have been widely utilized in Saint Lucia by the MOHW, the Internet has not, and clearly deserves more attention.

While the knowledge and attitude towards the HP screening test is considered to be good among HCWs, they were inclined to prefer cord blood (CB) screening. The reasons included the ease of doing the CB testing, faster turnaround time for results, lower cost, and greater availability of the materials needed for the test. They emphasized that the CB testing does not require specialized tools or equipment, since syringes are always readily available. On the other hand, the HP method required special-size lancets and filter paper, which were sometimes in short supply. This contributed to delays in sending the samples to Jamaica for testing. This was compounded by the fact that a sufficient number of samples had to be collected before they were sent to Jamaica to be analyzed.

### Recommendations

Information on the HP screening method for SCD should be provided to all women in the reproductive age group by HCWs, and in both the public and private health sectors. The Bureau of Health Education in the MOHW should be charged with the responsibility of developing targeted messages for pregnant women receiving antenatal care at wellness centers, through private practitioners, and at hospitals. HCWs should be trained or retrained in order to build their capacity to provide quality service to women in the reproductive age group. The MOHW’s strategic plan for health care should incorporate tools to monitor and evaluate outcomes of programs, such as the SCD one, that can contribute to improved maternal and child health.

### Conclusions

The HCWs’ knowledge of the HP screening method was high. However, there are other knowledge gaps that need attention. The mothers trust HCWs, and would accept the HP procedure irrespective of their knowledge of the test and any discomfort associated with this screening method. While these findings may indicate the likelihood of successful HP implementation, the health system needs to emphasize greater information-sharing. This will enable mothers to make informed decisions, instead of merely relying on their trust in the HCWs.

## Acknowledgments

Conducting this study would not have been possible without the invaluable cooperation and participation of the health care workers and the new mothers in the maternity wards of the three hospitals. Special thanks go to Deaina St. Marthe, Marcellina Williams, Jean Frederick, Tinisha Mitchel, and Curtis Jn. Charles for their assistance with data collection and data entry. The authors also wish to acknowledge the sterling contribution of the staff of the Epidemiology Unit of the Ministry of Health and Wellness, including Nahum Jn. Baptiste and Phil Leon.

## Funding.

Support for this study came from two sources: (1) the Pan American Health Organization and its grants to improve program delivery, which are supported by the Alliance for Health Policy and Systems Research (AHPSR), which is hosted by the World Health Organization, and (2) the Government of Saint Lucia, through the Ministry of Health and Wellness.

## Disclaimer.

Authors hold sole responsibility for the views expressed in the manuscript, which may not necessarily reflect the opinion or policy of the *RPSP/PAJPH* or PAHO.

## References

[1] 1. National Institutes of Health. Explore sickle cell disease. Available from: http://www.nhlbi.nih.gov/health/health-topics/topics/sca Accessed 15 December 2015.

[2] 2. Makani J, Cox SE, Soka D, Komba AN, Oruo J, Mwamtemi H, Mortality in sickle cell anemia in Africa: a prospective cohort study in Tanzania. PLoS One. 2011 Feb 1662e1469910.1371/journal. pone.0014699. 10.1371/journal.pone.0014699 PMC3040170 21358818

[3] 3. World Health Organization. Sickle cell disease and other haemoglobin disorders. Available from: http://www.who.int/mediacentre/factsheets/fs308/en/ Accessed 15 January 2016.

[4] 4. Central Statistics Office, Saint Lucia. Population and vital statistics. Available from: www.Stats.gov.lc Accessed 10 February 2017.

[5] 5. Ministry of Health and Wellness, Saint Lucia. Saint Lucia sickle cell programme audit. Castries: Ministry of Health and Wellness; 2014.

[6] 6. World Health Organization. Genes and human disease. Available from: http://www.who.int/genomics/public/geneticdiseases/en/index2.html Accessed 15 January 2016.

[7] 7. Nicholls SG. Southern KW. Parental decision-making and acceptance of newborn bloodspot screening: an exploratory study. PLoS One. 2013 Nov 12;8(11):e79441. doi: 10.1371/journal.pone.0079441. eCollection 2013. 10.1371/journal.pone.0079441 PMC3827133 24265771

[8] 8. Vansenne F, de Borgie C, Bouva MJ, Legdeur MA, van Zwieten R, Petrij F, Sickle cell disease in neonatal screening: identification of sickle cell trait. Ned Tijdschr Geneeskd. 2009:153:858–61. 19475864

